# BMP-4 and fetuin A in systemic sclerosis patients with or without calcinosis

**DOI:** 10.3389/fimmu.2024.1502324

**Published:** 2024-12-03

**Authors:** Francesco Demetrio Lofaro, Dilia Giuggioli, Susanna Bonacorsi, Martina Orlandi, Amelia Spinella, Marco De Pinto, Ottavio Secchi, Clodoveo Ferri, Federica Boraldi

**Affiliations:** ^1^ Dipartment of Life Sciences, University of Modena and Reggio Emilia, Modena, Italy; ^2^ Department of Maternal, Child and Adult Medical and Surgical Sciences, University of Modena and Reggio Emilia, Modena, Italy

**Keywords:** bone morphogenic protein, calcification, fetuin A, scleroderma, serum calcification propensity

## Abstract

**Introduction:**

Systemic sclerosis (SSc) is a connective tissue disease at the interface between inflammation and autoimmunity progressively leading to diffuse microvascular and fibrotic involvement of the skin and of multiple internal organs. Approximately, 20-40% of SSc patients suffer from cutaneous calcinosis, a debilitating manifestation due to calcium salt deposition in soft connective tissues, causing pain, ulceration, infection, and deformities, responsible of severe functional limitations. Pathomechanisms are poorly understood as well as markers/molecules capable to predict the risk of patients to develop calcinosis.

**Methods:**

An observational study was performed in 51 female patients, 25 with and 26 without calcinosis to compare clinical and laboratory parameters and to evaluate pro- and anti-calcifying circulating markers and the *in vitro* serum calcification potential (T50). Moreover, calcinosis samples were analyzed to characterize their mineral composition.

**Results and discussion:**

Data demonstrate statistically significant differences in the prevalence of clinical manifestations and ACA and Scl70 autoantibodies in SSc patient with calcinosis compared to those without calcinosis. In SSc patients with calcinosis, serum levels of BMP-4 are higher, fetuin A might be regarded as a potential circulating prognostic marker and a negative correlation was observed between T50 and the global score of clinical manifestations, suggesting a potential predictive role of pro- and anti-calcifying molecules in SSc patients. Furthermore, calcinosis samples were characterized by the co-existence of phosphate and carbonate minerals with different stability and solubility. Further investigations on circulating markers in larger patient cohorts, especially at the early stages and throughout the natural course of the disease, may clarify their pathogenetic role in the SSc-related cutaneous calcinosis.

## Introduction

Systemic sclerosis (SSc) is a connective tissue disease at the interface between inflammation and autoimmunity progressively leading to fibrosis of the skin and of multiple internal organs (1).

Calcinosis is a clinical complications occurring in 20-40% of SSc patients (2–5) being characterized by calcium salt deposition in skin and in subcutaneous tissues causing pain, ulceration, infection, and deformities causing severe functional limitations (6). The underlying mechanisms are still elusive, even if chronic inflammation, trauma, and vascular damage have been hypothesized (7). In the last years studies performed in unrelated diseases (*e.g.*, vascular calcification, chronic kidney disease, ectopic mineralization on genetic basis) demonstrated that pathological calcification is a complex process in which molecular (*e.g.*, fetuin A, osteopontin and BMP-2, -4) and/or different cellular mediators (*e.g.*, smooth muscle cells, circulating vascular progenitor cells and fibroblasts) may play a key role (8, 9).

However, the role of calcification regulatory proteins has been poorly investigated in the pathogenesis of SSc-related calcinosis (10).

Furthermore, the mineral phase of calcinosis has not yet been clearly defined due to contradictory results either describing calcinotic particles preferentially composed of hydroxyapatite (HAP), or of carbonate apatite (11, 12), that has a different stability and solubility compared to HAP.

Therefore, the present observational study aimed to compare clinical and laboratory parameters in SSc patients with (C group) or without (NC group) cutaneous calcinosis. Moreover, circulating markers and *in vitro* serum potential of calcification were evaluated in the two subgroups of patients. Finally, in a few SSc patients, the composition of calcium deposits was also investigated.

## Materials and methods

### Patients and clinical data

The present observational case control study included 25 SSc patients with cutaneous calcinosis (C group) and 26 without (NC group). All patients gave written informed consent, and the study was approved by the local Institutional Ethical Committee (protocol no. 275/16). The study was performed in adherence with the Declaration of Helsinki. Patients classified according the ACR/EULAR 2013 SSc criteria (13) were consecutively recruited at the Scleroderma Unit of the University-based Scleroderma Unit of the Policlinico of Modena, Italy, from March 2023 to October 2023. Moreover, to avoid comorbidities possibly involving the calcification process, SSc patients with diabetes, osteometabolic diseases, skin cancer and chronic comorbidities (*i.e.*, thyroid-parathyroid pathology, diabetes mellitus, alterations in phosphocalcic metabolism, chronic renal failure) were excluded.

The presence of cutaneous calcification was invariably confirmed by physical examination, prior imaging, or both (6).Baseline information including demographic data, clinical signs and symptoms, organ involvement, capillaroscopic pattern and current therapies were collected.

In all patients of group C calcium deposits were classified according to their shape and consistency on palpation (14). Digital calcinotic samples were obtained from the fingers of 7/25 patients (age range 48y–72y) using a scalpel in sterile conditions, according to standard procedures applied to lower patients’ pain and to partially restore mobility (15).

Routine laboratory analyses, including serum levels of calcium, phosphorus, magnesium, alkaline phosphatase (ALP), vitamin D, parathyroid hormone (PTH), and autoantibodies were performed in both patients’ subgroups.

A global score was specifically created to evaluate the total burden of clinical manifestations.

Clinical manifestations were evaluated in each patient as a “global score” (GS) resulting for the sum of the occurrence (+1) or absence (0) of telangiectasias, digital ulcers, diffuse subset, interstitial lung disease, PAH, artery hypertension, coronary artery disease, gastro-esophageal reflux disease, esophageal dysfunction, intestinal involvement, osteoporosis, renal involvement.

### Measurement of serum calcification propensity

Calcium and phosphate can combine *in vivo* to form amorphous primary calciprotein particles (CPP1), which can subsequently transform in secondary CPP (CPP2) containing crystalline calcium phosphate. The time required for half of the CPP1 to convert into CPP2 is known as T50, which reflects the capacity of serum to resist crystallization of calcium and phosphate (16).

T50 and CPP2 were measured in the serum samples collected from all SSc patients through venipuncture at the same moment of sampling for routine blood chemistry. The T50 and CPP2 were performed at Calciscon (AG, Nidau, Switzerland) according to Pasch and colleagues (16).

### Enzyme-linked immunosorbent assay

Serum levels of BMP-2 (ab277085), BMP-4 (ab231930), fetuin-A (ab269372), osteopontin (OPN) (ab269374), osteoprotegerin (OPG) (ab189580) and SPARC/osteonectin (ab220654) were assessed in a blinded manner using commercial kit according to manufacturer’s instructions.

### Scanning electron microscopy with energy-dispersive spectroscopy

Samples removed from the fingers of patients were firstly observed by SEM (Nova NanoSEM 450, FEI, Hillsboro, OR, USA) with a backscattered electron signal (BSE) to examine the morphology and the distribution of calcified areas. The analysis of the elements present in the calcinosis samples was performed by EDS, as already described (17).

### X-ray diffraction

The mineral phase present in the samples was evaluated by XRD. Spectra were recorded using a Bruker AXS D8 Advance spectrometer (Bruker AXS, MA, United States) with a step size of 0.02, at a scanning rate of 0.1–1.2 s in a 2θ range from 10 to 70. XRD pattern was analyzed using Origin Pro 2020 software (OriginLab).

### Raman spectroscopy

Raman spectra were collected with a micro-Raman system (LabRam HR Evolution, HORIBA Scientifc) using 785-nm laser excitation wavelength, 100 × objective (Olympus, numerical aperture, 0.9). The power of the incident laser was 100 mW. The typical spectral resolution was 4 cm^−1^ and 300 scans were applied. Spectra were corrected at baseline to suppress the luminescence background.

The instrument was daily aligned, and intensity calibrated using automated procedures implemented in the instrument start-up process.

The background correction used to reduce fluorescence signals was removed in LabSpec 6 (HORIBA Scientifc) using baseline correction by fitting and subtracting a polynomial function of the 4^th^ order to each spectrum.

The reference spectra of inorganic components were obtained from both literature data (18, 19) and the RRUFF database (https://rruff.info/).

### Statistical analyses

Statistical analyses were performed using GraphPad Prism version 9.0 (GraphPad Software, San Diego, CA, USA). Continuous variables with Gaussian distribution or with skewed distribution are presented as mean ± standard deviation and as median and interquartile range, respectively. Shapiro-Wilk test was applied to evaluate normal distribution of data. The variable comparisons were performed using parametric or no parametric tests for variables with Gaussian or skewed distribution, respectively. The chi-square test was used to compare categorical variables. Pearson correlation analysis was used to assess correlations between variables, and significant correlations were analyzed using linear regression. P values < 0.05 were considered significant.

## Results

### Patient characteristics

The demographic, clinical, and serological characteristics of patients with (C group) or without (NC group) calcinosis are shown in **Table 1**. No statistically significant differences in the epidemiological characteristics were observed among groups, with the exception of longer disease duration in the C group (**Table 1**). Some disease manifestations were recorded at higher prevalence in patients with calcinosis compared to those in the NC group, such as telangiectasias, systemic and artery pulmonary hypertension, gastro-oesophageal reflux, intestinal involvement (**Table 1**).

**Table 1:** Clinical and laboratory characteristics of SSc patients (pt) with (C group) or without calcinosis (NC group).

	NC group (26 pt)	C group (25 pt)	p value
Age (years), mean ± SD	63 ± 10	67 ± 13	n.s.
Female, n (%)	26 (100%)	25 (100%)	n.s.
Body mass index, mean ± SD	23 ± 2.9	21.6 ± 3.0	n.s.
Smoke history, n (%)	10 (38%)	8 (32%)	n.s.
SSc subtype			
-Limited (lcSSc)	18 (69%)	21 (84%)	n.s.
-Diffuse (dcSSc)	8 (31%)	4 (16%)	n.s.
Duration of Raynaud’s phenomenon (years), mean ± SD	18 ± 11	22 ± 13	n.s.
Disease duration at recruitment (years), mean ± SD	13 ± 8.0	20 ± 10	**0.0093**
Telangiectasias, n (%)	13 (50%)	23 (92%)	**0.0010**
Interstitial lung disease, n (%)	14 (54%)	13 (52%)	n.s.
Pulmonary arterial hypertension, n (%)	0 (0%)	5 (20%)	**0.0001**
Artery hypertension, n (%)	7 (27%)	14 (56%)	**0.0349**
Coronary artery disease, n (%)	0 (0%)	1 (0.4%)	n.s.
Hyperlipidemia, n (%)	15 (58%)	20 (80%)	n.s.
Gastro-oesophageal reflux disease, n (%)	11 (42%)	22 (88%)	**0.0006**
Osteoporosis, n (%)	11 (42%)	13 (52%)	n.s.
Oesophageal dysfunction, n (%)	12 (46%)	17 (68%)	n.s.
Intestinal involvement, n (%)	4 (15%)	10 (40%)	**0.0489**
Digital ulcers, n (%)	10 (38%)	17 (68%)	n.s.
Hyperlipidemia, n (%)	15 (58%)	20 (80%)	n.s.
Serum calcium (mg/dL) (8.5-10.5 mg/dL), mean ± SD	9.26 ± 0.27	9.28 ± 0.52	n.s.
Serum phosphate (mg/dL) (2.5-5.1 mg/dL), mean ± SD	3.83 ± 0.52	3.48 ± 0.43	n.s.
Serum Mg (mg/dL) (1,6-2,6 mg/dl), median (IQR)	1.90 (1.80 – 2.00)	1.90 (1.80-2.05)	n.s.
25-hydroxyvitamin D (ng/mL), mean ± SD	37.5 ± 10.4	36.3 ± 13.6	n.s.
Parathyroid hormone (pg/mL) (6.5 - 36.8 pg/mL) median (IQR)	30.7 (23.2–46.7)	30.60 (25.2–43.2)	n.s.
Alkaline phosphatase unit/L (38 - 126 unit/L), median (IQR)	67.0 (54.5–73.0)	62.0 (46.5–73.0)	n.s.
HDL (mg/dL) > 43, mean ± SD	62.0 ± 14.1	61.0 ± 16.0	n.s.
LDL (mg/dL) < 115, mean ± SD	120 ± 21.6	123 ± 33	n.s.
Albumin g/dL (3.5 - 5.0 g/dL), mean ± SD	4.01 ± 0.23	3.94 ± 0.41	n.s.
C-reactive protein mg/dL (0 - 0.7 mg/dL), median (IQR)	0.30 (0.27–0.52)	0.30 (0.2-0.85)	n.s.
eGFR (mL/min/1.73 m2) < 60 n (%)	5 (19%)	2 (8%)	n.s.
Global score, mean ± SD	3.85 ± 2.20	5.64 ± 2.28	**0.0064**
Treatment:			
Vitamin D use, n (%)	26 (100%)	25 (100%)	n.s.
Phosphate binder use, n (%)	0 (0%)	0 (0%)	n.s.
Lipid-lowering medication use, n (%)	7 (27%)	11 (44%)	n.s.
Bisphosphonate use, n (%)	2 (8%)	4 (16%)	n.s.
Denosumab use, n (%)	2 (8%)	5 (20%)	n.s.

Bold significant values. n.s., no significant.

In terms of antibody profiles, the percentage of SSc patients positive for anti-centromere (ACA) and anti-topoisomerase (Scl70) autoantibodies differed significantly between NC and C groups. Specifically, a higher percentage of patients in the C group were positive for ACA compared to the NC group (72% *vs* 35%, p=0.0003), contrarily to that observed for serum anti-Scl70 (16% *vs* 54%, p=0.0047). No significant differences between the two groups were observed for anti-nuclear, anti-RNA polymerase III, and SSA-SSB antibodies.

Interesting, no significant differences were found regarding both the capillaroscopic pattern and features.

The GS was significantly higher in C than in the NC group (p= 0.0064) (**Table 1**).

### Circulating promoters and inhibitors of calcinosis

The serum levels of molecules known to favor (*i.e.*, BMP-2, BMP-4 and SPARC) or to inhibit (*i.e.*, OPN, fetuin A and OPG) the calcification process have been investigated in the two groups of patients.

**Figure 1:**
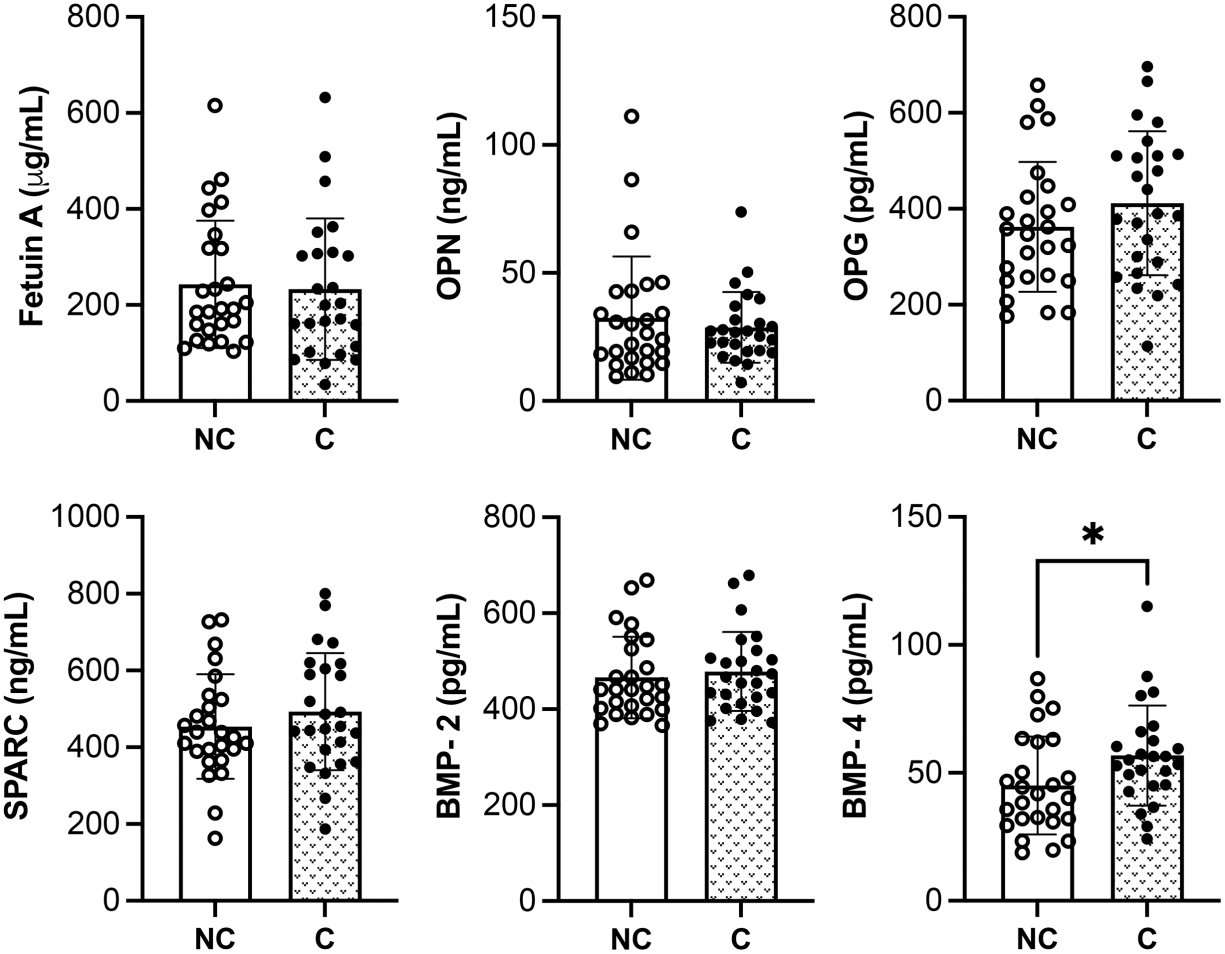
Quantitative analysis of pro- and anti-calcified molecules in SSc patients with (C) or without calcinosis (NC). The results are shown as mean ± standard deviation (n= 26 NC patients, n= 25 C patients). *p<0.05. OPN, osteopontin; OPG, osteoprotegerin; SPARC, osteonectin; BMP, bone morphogenetic protein.

**Figure 1** shows that serum levels of both pro- and anti-calcified molecules are not significantly different between NC and C groups, except for BMP-4.

A bivariate correlation analysis was performed between these markers and the GS within the same group of patients. Fetuin A inversely correlated with GS in the C group (**Table 2**).

**Table 2:** Correlation of clinical manifestations (global score) with serum anti- and pro-calcifying molecules in SSc patients with (C) and without (NC) calcinosis.

Variables	Correlation with global score			
	NC group		C group	
	*r*	P value	*r*	P value
**Fetuin A**	0.021	0.92	**-0.400**	**0.04**
**BMP-2**	-0.034	0.87	-0.026	0.90
**BMP-4**	-0.172	0.40	0.017	0.94
**OPN**	0.182	0.42	0.041	0.85
**OPG**	0.180	0.46	0.227	0.27
**SPARC**	-0.176	0.39	0.024	0.91

Pearson correlation analysis was used. BMP, bone morphogenetic protein; OPN, osteopontin; OPG, osteoprotegerin; SPARC, osteonectin.

Significant correlation is in bold.

### Serum calcification propensity

**Figure 2:**
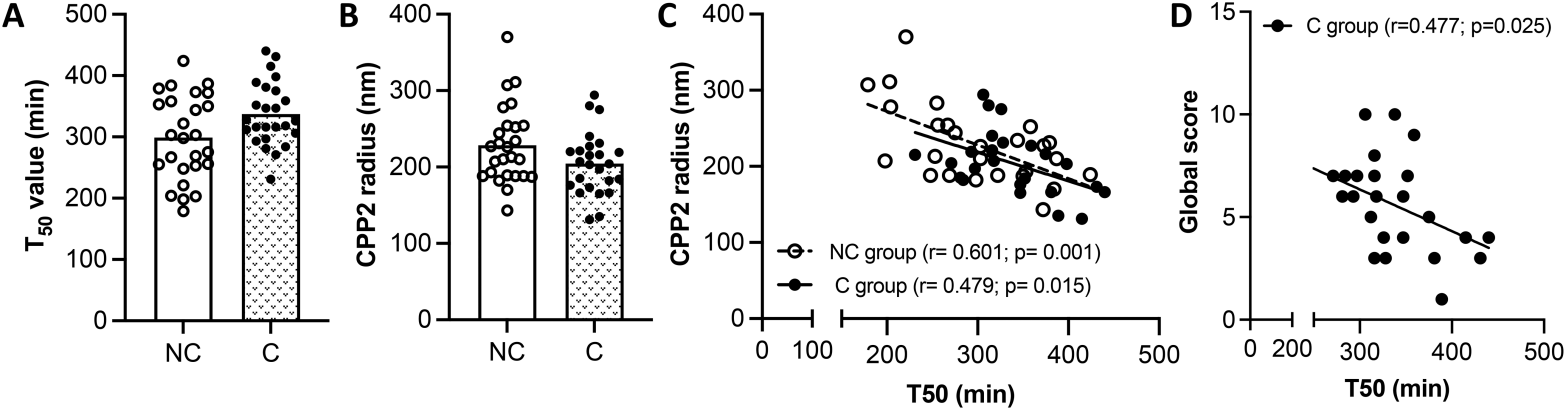
Measurement of serum calcification propensity. **(A, B)** Half-maximal time (T50), and secondary calciprotein particles size (CPP2) in SSc patients with (C group) and without (NC group) calcinosis. The results are shown as mean ± standard deviation (n= 26 NC patients, n=25 C patients). **(C)** Linear regression between T50 and CPP2 size in NC and C groups. **(D)** Linear regression between T50 and global score in NC and C patient groups.

**Figures 2A, B** shows that T50 value and CPP2 size are similar between the two groups. These two parameters negatively correlated with each other in both groups (*r*_NC group_= -0.601, p= 0.001 and *r*_C group_= -0.479, p= 0.015). A linear relationship exists between the two variables in both patient groups (**Figure 2C**). Whereas no correlation was found between T50 and GS in the NC patient group, a significant negative correlation was observed in the C patient group, showing a linear relationship (**Figure 2D**). CPP2 size did not correlate with the GS in both NC and C patient groups (*r*_NC group_= -0.236, p= 0.246 and *r*_C group_= 0.384, p= 0.058).

**Figure 3:**
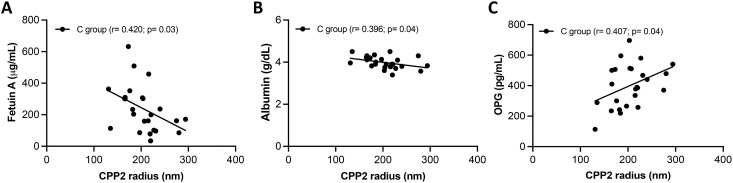
Associations between secondary calciprotein particles size (CPP2) and serum inhibitors of calcification. A linear relationship was found between CPP2 size and fetuin A **(A)**, albumin **(B)** and, and osteoprotegerin (OPG) **(C)** in SSc patient with calcinosis (C group).

It is known that T50 and CPP2 can be influenced by several factors such as fetuin-A, phosphate, calcium, magnesium and albumin (16). Correlation between T50 value or CPP2 size with markers of mineral metabolism (*i.e.*, Ca, P, Mg, ALP and PTH) and with inhibitors of mineralization (*i.e.*, serum albumin, fetuin A and OPG) were evaluated within the same group of patients. No correlations with T50 were found in NC and in the C group (data not shown). CPP2 radius did not correlate with any molecule analyzed in the NC group (data not shown); on the contrary, CPP2 correlated negatively with fetuin A (r= -0.428, p= 0.03) and serum albumin (r= - 0.396, p= 0.04), but positively with OPG (r= 0.399, p= 0.04) in the C group. A linear relationship was observed in all cases (**Figures 3A–C**).

### Analysis of calcinosis samples

**Figure 4:**
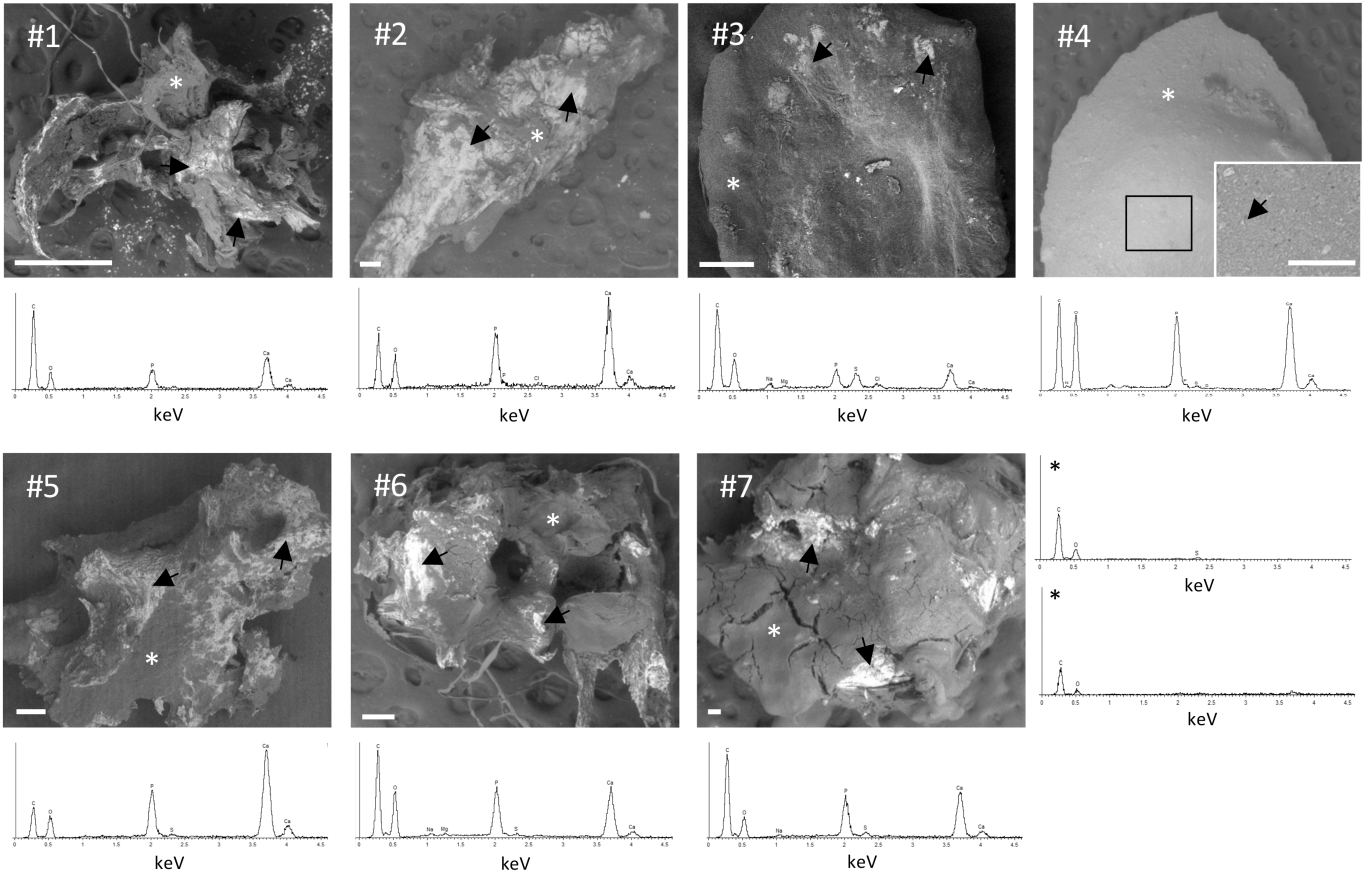
Scanning electron microscopy (SEM)/energy dispersive spectroscopy (EDS) analysis of calcinosis samples. Unfixed and unstained calcinosis samples were observed by SEM. Representative EDS spectra obtained in areas marked with an arrow show peaks of P and Ca. Two representative EDS spectra in areas marked with asterisk (*) display the absence of peaks of P and Ca. Scale bar= 100 µm.

Calcinosis samples were mainly characterized by dense and compact material except for the sample #4, that appears as a toothpaste-like fluid. The size of crystals was very heterogeneous depending on the samples (**Figure 4**).

Small spherical mineral particles were concentrated (#3) or spread (#4) into the samples, whereas in other specimens (*i.e.*, #1, #2, #5, #6 and #7) calcification was in form of small spots with a tendency to fuse into large mineralized areas with a jagged surface.

Element analyses were performed by EDS on the entire surface of the samples. P and Ca were not detected in the absence of mineralization (**Figure 4**, last panel). In calcified areas, high peaks of P and Ca were always observed, whereas ions such as Na, Cl, S, and Mg were detected only in some samples (**Figure 4**), partially or totally substituting ions present in calcium-phosphate minerals. These results indicate that P and Ca deposition is not homogeneous in calcinosis samples.

EDS spectra of calcinotic particles allow to obtain information about the elements present in the sample, but do not provide data on the chemical composition and on the crystals’ properties.

**Figure 5:**
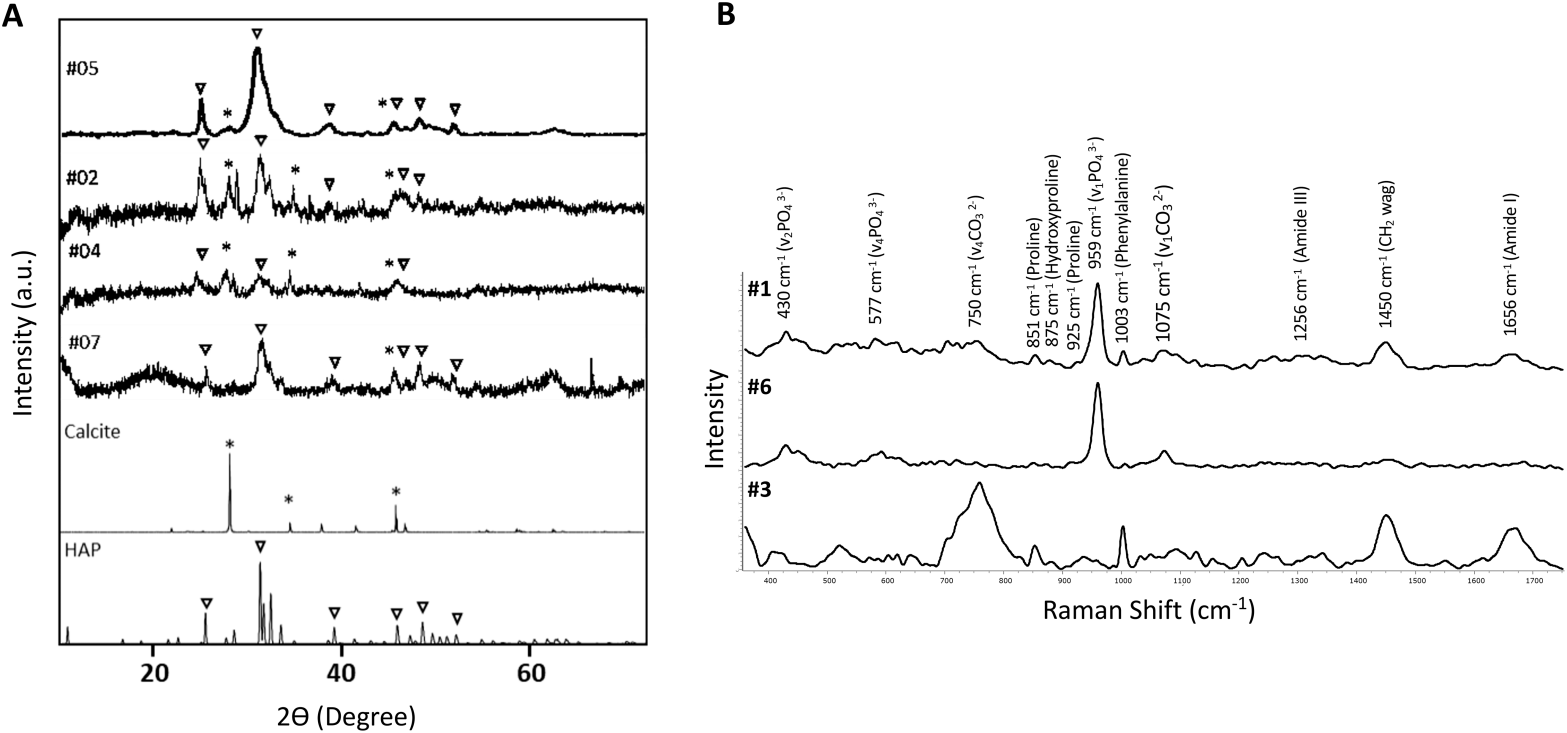
X-ray diffraction pattern and Raman spectrum of calcinosis samples. **(A)** X-ray diffraction patterns of minerals in samples #2, #4, #5 and #7. Symbol ∇ corresponds to hydroxyapatite (HAP) and * to calcite. HAP (JCPDS: 96-900-1234) and calcite (JCPDS:96-901-5836) reference patterns are shown at the bottom of the figure. **(B)** RAMAN spectrum of samples #1, #3 and #6.

By X-ray scattering (XRD), samples #2, #4, #5 and #7 exhibit peaks characteristic of HAP located at 2θ = 25.92° (002), 31.79° (121), 39.73° (310), 46.69° (222), 49.52° (123), 53.19° (004) and of calcite located at 2θ = 29.27 (104), 47.07 (018) and 48.34 (116) (**Figure 5A**). Three out of seven samples (#1, #3, #6) were characterized by a higher proportion of organic components compared to the inorganic ones, thus making it challenging to accurately identify the mineral phase through XRD analysis. Therefore, these samples underwent analysis by Raman spectroscopy. **Figure 5B** shows peaks at 430 cm^-1^ and 577 cm^-1^, corresponding to symmetric bending mode of v_2_ PO_4_^3-^ and the triply degenerate asymmetric bending vibration of v_4_ PO_4_^3-^, respectively. A prominent peak is observed at 959 cm^-1^, ascribed to v_1_ fundamental vibration mode arising from PO_4_^3-^ group. Moreover, a peak at 750 cm^-1^ and at 1075 cm^1-^ (corresponding to v_4_ and symmetric vibration mode v_1_ from carbonate, respectively) are present in all samples, in particular in sample #3. The B-type carbonate presence indicates, therefore, the partial substitution of PO_4_^3-^ with CO_3_^2^. CO_3_^2-^ carbonate anions can partially substitute also hydroxide ions (OH) (A-type), however A-type, characterized by two peaks at 1106 cm^1-^ and 1018 cm^1-^, was not detected in any samples. These findings indicate the co-existence of phosphate and carbonate minerals in the same calcinosis sample.

In addition, peaks are observed at 851 cm^−1^ (C–C proline, hydroxyproline), 1003 cm^−1^ (phenylalanine ring breathing); 1250-1350 cm^−1^ (amide III); 1452 cm^−1^ (H–C–H union); 1600-1800 cm^−1^ (C=O stretching, amide I), indicating the presence of organic matrix (20, 21).

## Discussion

Calcinosis is a manifestation of SSc characterized by the deposition of calcium minerals in the skin and in subcutaneous tissues (4).

Some Authors report an association between calcinosis either with dcSSc (22) or lcSSc (10), whereas other studies (23), including this one, did not found differences in the prevalence of calcinosis between SSc cutaneous subtypes. To explain these discrepancies, it has been suggested that different results can depend on ethnicity (*e.g.*, Caucasians *vs* African-American) and/or on geographic regions (4, 24). Moreover, as already reported (25), calcinosis occurs after several years (*i.e.*, 7-10 years) from SSc diagnosis.

Regarding internal organ involvement, patients with calcinosis develop more frequently arterial hypertension, gastro-esophageal reflux disease, intestinal involvement, and pulmonary arterial hypertension (PAH). This observation confirms previous data showing that calcinosis is associated with a more severe disease course (26). Interestingly, PAH is a serious complication of SSc affecting 8-15% of patients (27) and has been related to inflammation and calcification (28). In agreement with data from the literature, our patients show a significant association between PAH and calcinosis. It is worth noting that 20% of our SSc patients in the C group, but none in NC group, were complicated by PAH, although it cannot be excluded a possible contribution of longer disease duration characterizing this patient subgroup. Several studies have underlined some clinical characteristics and serological markers associated with the development of PAH in SSc patients, such as the presence of calcinosis, or telangiectasias, or lcSSc, and/or serum ACA (26, 29–32). In our study, both ACA seropositivity and telangiectasias are significantly more frequent in patients with calcinosis compared to those without.

In several pathological contexts, ectopic calcification has been shown to be associated with a systemic imbalance between pro- and anti-calcifying molecules (33). Since, scattered and sometimes contradictory data are present for SSc patients, we have investigated several circulating calcification-related molecules in both C and NC groups of patients.

Fetuin A is a systemic inhibitor of calcification and is known to protect tissues from inflammation-related damage. No difference was observed between C and NC patients, in contrast with data from Belloli and coworkers who found fetuin A to be significantly lower in serum of SSc patients with calcinosis compared to those without calcinosis (10). This discrepancy could be attributed to the smaller randomly selected sample size of patients analyzed in the previous study (10). Moreover, we cannot exclude that the levels of fetuin A might differ between the two groups in the early phases of SSc. For instance, in chronic kidney disease (CKD) patients, it has been suggested that fetuin A may have a protective role being upregulated in the early stages of the disease, whereas severe or prolonged exposure to a pro-inflammatory and/or pro-calcific environment may eventually lead to low levels due to decreased production and/or increased consumption (34). As mentioned above, calcinosis usually occurs after a prolonged disease course (*i.e.*, 7-10 years after SSc diagnosis).

Indeed, several studies have highlighted an association between fetuin A levels and the severity of different pathologic conditions (*e.g.*, chronic obstructive pulmonary disease, coronary calcification; CKD; calcific aortic valve diseases, aortic stiffness) (35–37). Therefore, the inverse correlation found between fetuin A levels and the global score in the C group of patients may suggest that lower fetuin A is associated with an increased number of clinical manifestations.

Osteopontin (OPN), a highly negatively charged secreted protein, regulates several processes including apoptosis, bone metabolism, inflammatory response, fibrosis. It has been also found in sites of ectopic calcification (*e.g.*, renal stones, aortic stenosis) suggesting that it can interact with crystal surfaces, and regulate the mineral process (38). No changes were observed in the present study, although high levels of serum/plasma OPN were previously found in SSc patients or in SSc patients with interstitial lung disease, however, in these studies SSc patients were compared with healthy controls without considering the presence of calcinosis (39–41).

Osteoprotegerin (OPG) is a soluble decoy receptor for the receptor activator of nuclear factor-κB ligand (RANKL), which stimulates osteoclastic bone resorption (42). The literature on OPG levels in SSc is quite heterogeneous. For instance, one study reported higher OPG levels in lcSSc patients compared to healthy subjects (43), while Tayalan Ali and collaborators (44) found similar OPG levels in both SSc patients and healthy subjects. In the present study, OPG levels were similar in C and NC patients. To our knowledge, only one study showed higher OPG levels comparing SSc patients with and without calcinosis (45). Differences between our results and previous findings could be due to the technical procedures utilized, as suggested by the detection range, and/or by the fact that in the previous study males and females were not separated, despite the known sex-dependent variation in OPG levels (higher in females than in males) (45).

SPARC/osteonectin is a protein with collagen type I-binding domain and hydroxyapatite-binding sites (46, 47) that may be involved in both the initial and the progressive stages of the calcification process (48, 49). This molecule was previously found to be significantly increased in the dermis of lcSSc patients with calcinosis compared to those without calcinosis (50) and in the plasma/fibroblasts from SSc patients compared to healthy subjects (51, 52). The discrepancies between our findings showing no differences in the two groups of patients and those reported in the literature may be related to: i) different types of bio-samples analyzed (*i.e.*, dermis/fibroblasts/plasma *vs* serum) and/or on ii) differences in the studied population, *i.e.*, we firstly stratified SSc patients based on the presence or the absence of calcinosis, whereas in other studies, patients with or without calcinosis were selected within one SSc cutaneous subtype (*i.e.*, lcSSc).

Bone morphogenic proteins –2 and -4 (BMP-2 and BMP-4) play a crucial role in vascular calcification by promoting osteogenic activation of vascular cells (53) and have also been implicated in cutaneous calcification (54) and inflammation (55).

Currently, there are no data in the literature on BMP-2 and BMP-4 in SSc patients. Our results indicate no changes for BMP-2, but higher BMP-4 levels in the C group. BMP-4 has been extensively investigated as a promoter of calcification either in the vascular system (53) and in the skin (54). Besides its role as pro-osteogenic signaling inducer, BMP-4 is also related to inflammation and vascular damage, inducing endothelial dysfunction through oxidative stress (56). Since overexpression of BMP-4 in endothelial cells enhances vascular remodeling in pulmonary hypertension (57) it cannot be excluded that higher serum levels of BMP-4 can contribute to the vascular abnormalities frequently observed in the C group (*e.g.*, telangiectasia, PAH and arterial hypertension). This may, in turn, accelerate the need of remodeling and renewal of endothelial cells, potentially leading to a shift towards a pro-osteogenic phenotype (58) and underlines the potential role of BMP-4 in SSc calcinosis.

Under certain conditions, Ca, P and specific serum proteins (*e.g.*, fetuin A and albumin) can aggregate to form amorphous, soluble Ca–P particles (CPP1). These particles can subsequently transform into larger and crystalline calciprotein particles (CPP2) and the half-maximal time of transformation from CPP1 to CPP2 is known as T50. A lower T50 indicates a faster conversion from CPP1 to CPP2, and this has been associated, for example, with cardiovascular mortality, and with progressive aortic stiffening (59, 60). In our study, we did not observe significant differences of T50 and CPP2 between C and NC groups. However, we revealed a correlation between T50 and the global score, as well as between CCP2 and circulating inhibitors of calcification.

Since its introduction in 2012 (16), T50 test has been evaluated in both healthy subjects and in various patient cohorts (*e.g.*, diabetic, hemodialysis, Pseudoxanthoma elasticum patients) (61–65) demonstrating that lower T50 values could predict all-cause and cardiovascular mortality in hemodialyzed patients, in those with CKD or with diabetes. Furthermore, some studies have proposed the use of T50 value as a surrogate marker of arterial calcification or as biomarker of disease severity (65). Interestingly, a negative correlation between T50 and global score was found in the C group. Although further studies with a larger number of SSc patients are necessary to confirm this result, T50 could be an important biomarker in SSc, potentially useful for risk stratification and management of the SSc patients with calcinosis.

Interestingly, in the C group we found that fetuin A and albumin negatively correlated with CCP2, consistently with the inhibitory role of fetuin A (66, 67) and of albumin (68) (69, 70) in preventing the growth and the aggregation of CPP2 thereby hindering their precipitation.

In addition, in the C group, larger CPP2 radius correlated with higher serum OPG. These results are akin to those obtained in hemodialysis patients and in pre-dialysis CKD patients (69, 71). However, further studies are needed to clarify the role of OPG in influencing the size of CPP2.

Ectopic calcification is generally associated with deposition and progressive accumulation of insoluble HAP, however, several studies demonstrated that different type of crystals with a different solubility are present in mineralization sites (72). Present results highlight that calcinosis, even in the same SSc patient, is characterized by a mixture of carbonate and phosphate minerals including calcite and HAP, respectively. It is important to note that analyses were performed on calcinosis samples removed from patients and not on isolated crystals. Since X-ray analysis is not capable to detect crystals that constitute less than 5% of the sample’s weight (73), we cannot exclude the additional presence of other minerals further increasing the complexity of calcinosis samples. Previous studies described SSc calcification composed solely of HAP or of B type carbonated apatite (11, 12, 74, 75). These discrepancies can be explained by the fact that calcification is a complex, non-linear process, and that mineral characteristics (*i.e.*, structure, composition and morphology) depend on several factors such as ion concentration, temperature, pH value and location (76–79). Some *in vitro* and *in vivo* studies report that a range of intermediate phosphates (*e.g.*, carbonated apatite, octacalcium phosphate) are formed before transforming into HAP (80–82), even though Thomson and collaborators suggested that hydroxyapatite is formed directly on cholesterol-containing droplets suggesting the absence of intermediate phases (83). The demonstration that different types of mineral deposits are present in SSc calcinosis may lead to the possibility of selecting the most appropriate treatment to improve the solubility of mineral components and to decrease extraosseous calcification. The differences observed among various samples could be due to changes in the composition/organization of the organic matrix. It is well known that aging, oxygen availability, mechanical stress, for example, can modify the characteristics of the extracellular matrix favoring the development/progression of pathological mineralization (84–86). Therefore, a thorough study of both the mineral and the organic components will provide a better understanding of the mechanisms leading to the development of calcinosis.

While the present study indicates some molecules that can correlate with calcinosis in SSc patients, it comes with some limitations such as: i) the relatively small number of patients in both subgroups; ii) the lack of data in the early phases of the disease; iii) the absence of male patients; iv) the difficulty to recruit patients in a fairly homogeneous phase of the disease.

Although these potential biases should be kept in mind to draw general conclusions, in summary, present data allow: i) to find statistically significant differences in the prevalence of clinical manifestations (*i.e.*, telangiectasias, pulmonary arterial hypertension, artery hypertension, gastro-oesophageal reflux disease, intestinal involvement) and laboratory tests (*i.e.*, ACA and Scl70 autoantibodies) in SSc patient with calcinosis compared to those without calcinosis; ii) to highlight higher serum levels of BMP-4 in SSc patients with calcinosis; iii) to identify fetuin A as potential circulating prognostic marker in SSc patients with calcinosis; iv) to underline a higher global score in C patient group; v) to show negative correlation between T50 and global score in patients with calcinosis; vi) to demonstrate the heterogenous composition of the mineral component in calcinosis samples.

The present data may pave the way for future studies on larger cohorts of patients. Moreover, measuring pro- and anti-calcifying molecules at the time of SSc diagnosis and during the clinical long-term follow-up in patients who will do or do not develop calcinosis might clarify their pathogenetic role.

## Data Availability

The original contributions presented in the study are included in the article/supplementary material. Further inquiries can be directed to the corresponding authors.
